# Biofilms of a *Bacillus subtilis* Hospital Isolate Protect *Staphylococcus aureus* from Biocide Action

**DOI:** 10.1371/journal.pone.0044506

**Published:** 2012-09-04

**Authors:** Arnaud Bridier, Maria del Pilar Sanchez-Vizuete, Dominique Le Coq, Stéphane Aymerich, Thierry Meylheuc, Jean-Yves Maillard, Vincent Thomas, Florence Dubois-Brissonnet, Romain Briandet

**Affiliations:** 1 INRA, UMR 1319 MICALIS, Jouy-en-Josas, France; 2 AgroParisTech, UMR MICALIS, Jouy-en-Josas, France; 3 CNRS, Jouy-en-Josas, France; 4 Welsh School of Pharmacy, Cardiff University, Cardiff, United Kingdom; 5 STERIS, Fontenay aux Roses, France; Institut Pasteur, France

## Abstract

The development of a biofilm constitutes a survival strategy by providing bacteria a protective environment safe from stresses such as microbicide action and can thus lead to important health-care problems. In this study, biofilm resistance of a *Bacillus subtilis* strain (called hereafter ND_medical_) recently isolated from endoscope washer-disinfectors to peracetic acid was investigated and its ability to protect the pathogen *Staphylococcus aureus* in mixed biofilms was evaluated. Biocide action within *Bacillus subtilis* biofilms was visualised in real time using a non-invasive 4D confocal imaging method. The resistance of single species and mixed biofilms to peracetic acid was quantified using standard plate counting methods and their architecture was explored using confocal imaging and electronic microscopy. The results showed that the ND_medical_ strain demonstrates the ability to make very large amount of biofilm together with hyper-resistance to the concentration of PAA used in many formulations (3500 ppm). Evidences strongly suggest that the enhanced resistance of the ND_medical_ strain was related to the specific three-dimensional structure of the biofilm and the large amount of the extracellular matrix produced which can hinder the penetration of peracetic acid. When grown in mixed biofilm with *Staphylococcus aureus*, the ND_medical_ strain demonstrated the ability to protect the pathogen from PAA action, thus enabling its persistence in the environment. This work points out the ability of bacteria to adapt to an extremely hostile environment, and the necessity of considering multi-organism ecosystems instead of single species model to decipher the mechanisms of biofilm resistance to antimicrobials agents.

## Introduction

In response to environmental conditions, bacteria have developed different strategies to adapt and survive. The formation of multicellular communities known as biofilms is one such strategy, which is associated with increased bacterial resistance to environmental stress accompanying antimicrobial treatments [1], [2]. It has indeed been shown that non-lethal concentrations of antibiotics or disinfectants can stimulate biofilm formation [3], [4] which constitutes a defensive response to the toxic effects of the biocides. Mechanisms involved in the resistance of biofilms remain unclear but appear to result from multifactorial processes related to the architecture of the edifice and the phenotypic features of the cells. The presence of a protective extracellular matrix via the expression of specific genes in the biofilm, the metabolic state of cells due to microenvironmental conditions have all been identified as playing a role in biofilm resistance to antimicrobial agents [5]. In the clinical settings, biofilms promote infection through, for example, the persistence of bacteria on medical devices and thus can act as reservoir for pathogens [6], [7]. It has been estimated that 65% of nosocomial infections directly involve biofilms [8]. Of all medical devices used, flexible endoscopes are among the most frequently associated with outbreaks of infectious organisms [9], [10], [11], [12], [13]. Indeed, some studies have reported the presence of persistent bacteria on endoscopes despite cleaning and disinfection procedures [14], [15]. Interestingly, a viable strain of *B. subtilis* was recently isolated from an endoscope washer-disinfector after high level disinfection with chlorine dioxide [16]. The authors showed that this isolate had developed cross-resistance against other oxidising agents using a standard suspension test. Recently, we showed that this stable isolate was able to produce a thick immersed biofilm with specific protruding structures [17]. In this work, we have now investigated the resistance of this strain in biofilm to peracetic acid (PAA), an oxidizing agent commonly used in formulations used for endoscope disinfection. In addition, we examined the ability of the ND_medical_ strain to protect *Staphylococcus aureus*, a pathogen responsible of a large number of hospital acquired infections [18], from biocidal action in a mixed biofilm.

## Materials and Methods

### Bacterial Strains and Growth Conditions

The bacterial strains used in this study are listed in Table 1. The GFP-carrying *Bacillus subtilis* ND_medical_ strain was obtained by transforming *B. subtilis* ND_medical_ to spectinomycin resistance with chromosomal DNA of strain GM2812 [17], thus placing in the *amyE* locus the *gfp (mut2)* gene under the control of the IPTG-regulated *P_hyperspank_* promoter. Transformation was performed according to standard procedures and the transformants were selected on Luria-Bertani (LB, Sigma, France) plates supplemented with spectinomycin at 100 µg/ml. Before each experiment, bacteria were subcultured twice in Tryptone Soya Broth (TSB, BioMérieux, France). When necessary, IPTG at a final concentration of 200 µM was added to the medium to induce GFP production, and antibiotics at the following concentrations: spectinomycin, 100 µg/ml; erythromycin, 3 µg/ml.

**Table 1:** Strains used in this study.

Strain	Relevant genotype or description a	Reference or construction
*B. subtilis* 168	trpC2	Bacillus Genetic Stock Center
*B. subtilis* 168 GFP	amyE::Phyperspank-gfp (specR)	[17]
*B. subtilis* ND_medical_	Freshly isolated from endoscope washer-disinfectors	[16]
*B. subtilis* ND_medical_ GFP	amyE::Phyperspank-gfp (specR)	*B. subtilis* GM2812 [17] ND_medical_
*S. aureus* AH478	Strain RN4220 with a plasmid harbouring a mCherry gene (eryR)	[48]

a *spec* and *ery* stand for spectinomycin and erythromycin resistance markers respectively.

### Biofilms Architecture

#### Confocal Laser Scanning Microscopy (CLSM)

Biofilms were formed in polystyrene 96-well microtiter plates with a µclear® base (Greiner Bio-one, France) enabling high-resolution fluorescence imaging as previously described [19]. For single species biofilms, 250 µl of an overnight culture in TSB adjusted to an OD_600nm_ of 0.02 were added to the wells of the microtiter plate. For mixed species biofilm, overnight cultures in TSB of *S. aureus* AH478 mCherry and the *B. subtilis* GFP strains were adjusted to an OD_600nm_ of 0.02 in TSB and mixed at a ratio of 1∶2. The microtiter plate was then kept at 30°C for 90 min to enable bacteria to attach to the bottom of the wells. After this adhesion step, the wells were rinsed with TSB to eliminate non-adherent bacteria and then refilled with 250 µl sterile TSB. The microtiter plate was then incubated for 24 h at 30°C to allow biofilm development. After development, biofilms were rinsed with 150 mM NaCl and series of xy images with a z-step of 1 µm were then acquired to explore the three-dimensional structure of the biofilms. Both single and mixed biofilms were scanned using an excitation wavelength of 458 nm (argon laser, 25% intensity) and 543 nm (helium-neon laser, 50% intensity), with emission wavelengths collected from 480 to 530 nm and from 580 to 700 nm respectively for GFP and mCherry fluorescence. Three-dimensional projections of biofilm structures were reconstructed using the Easy 3D function of the IMARIS 7.0 software (Bitplane, Switzerland) directly from xyz images series.

#### Scanning Electronic Microscopy (SEM)

For SEM observations, *B. subtilis* biofilms were prepared by immersing coupons in the wells of a 24-well polystyrene plate containing bacterial suspensions adjusted to an OD_600nm_ of 0.02. After 90 min of adhesion, coupons were rinsed and wells were refilled with 1 ml of TSB. The plate was then incubated at 30°C for 24 h. After development, biofilms were rinsed with 150 mM NaCl and attached bacterial cells were fixed in a solution containing 2.5% glutaraldehyde, 0.1 M sodium cacodylate (pH 7.4), 0.075% ruthenium red and 0.015% Alcian blue for 20 h. Ruthenium red and Alcian blue were added to the fixing solution because it have been showed that their presence could promote the visualization of extrapolymeric substances such as bofilm matrix components [20], [21]. Samples were then washed three times for 10 min with a solution containing 0.1 M sodium cacodylate, 0.075% ruthenium red and 0.015% Alcian blue before being transferred into 50% ethanol. Samples were progressively dehydrated by passage through a graded series of ethanol solutions from 50% to 100%. Finally, the samples were critical point dehydrated (Emitech K850, UK) using carbon dioxide as the transition fluid and coated with gold-palladium in an automatic sputter coater (Polaron SC7640, UK). Scanning electron microscopy Quanta 650 FEG-SEM (FEI, Japan) was used to observe the samples.

#### Congo red indicator assay on macrocolonies

The morphology of macrocolonies formed by both *B. subtilis* strains and their Congo Red binding properties were tested using indicator agar plates. Experimentally, 5 µl of an overnight culture in TSB were inoculated on TS agar (1.5%) containing 40 µg/ml Congo Red (Sigma-Aldrich, France). Plates were then incubated for 72 h at 30°C.

#### Determination of the sugar and protein contents of the biofilm matrix

The protein and sugar levels of the matrix of *B. subtilis* 168 and ND_medical_ biofilms were determined after 24 h of development in TSB. Biofilms were rinsed in distilled water and recovered from the microtiter plate by scraping the bottom of the wells with tips and aspirating and expelling the suspension at least 10 times. The suspensions were then vortexed during 30 seconds before being centrifuged at 4000 g during 20 minutes. After, the supernatants were transferred in sterile tubes and vortexed 30 seconds before being keeped at 20°C until biochemical assays were performed. Protein contents were evaluated using the Bradford assay [22] with bovine serum albumin as the standard. Sugar levels were determined by using the phenol-sulfuric acid assay procedure [23] with glucose as the standard. Each experiment was performed in triplicate on three independent biofilm extractions.

### Biocide Treatments

#### Biocide testing

After development, biofilms in the microtiter plates were rinsed and the wells refilled with 100 µl of TSB. Then, 100 µl of peracetic acid (PAA) (32 wt. % in dilute acetic acid (Sigma-Aldrich, France)) at 7000 ppm were then added in the wells to obtain a final concentration of 3500 ppm. After 5 min of contact, biocide solutions were gently removed and wells were refilled with a quenching solution (3 g/L L-α-phosphatidyl cholin, 30 g/L Tween 80, 5 g/L sodium thiosulfate, 1 g/L L-histidine, 30 g/L saponine) and left for 5 min to stop biocide action. The bottom of the wells was then scraped with tips and the suspension aspirated and expelled many times to detach and recover survivor cells from biofilms. The suspensions were then serially diluted in 150 mM NaCl and plated on Tryptone Soya Agar (TSA, Biomérieux, France) before being incubated at 30°C for 24–48 h.

To determine the sensitivity of *B. subtilis* ND_medical_ cells to PAA following disruption of the biofilm, biofilms were rinsed with TSB and cells were recovered by scraping the bottom of the wells with tips and aspirating and expelling many times the suspension. The recovered cells were mixed with 150 mM NaCl containing glass beads and suspension vortexed for 30 s to separate cells and extracellular matrix. The suspension was then washed after centrifugation (7000 g, 10 min, 20°C) and adjusted to 10^8^ CFU/ml in 150 mM NaCl. In parallel, planktonic bacteria were harvested from a 24 h culture in TSB at 30°C by centrifugation (7000 g, 10 min, 20°C) followed vortexing with glass beads (0.5 mm of diameter) for 30 s and then washed in 150 mM NaCl before also adjusting to 10^8 ^CFU/mL. Peracetic acid activity was then evaluated according to the standard European NF EN 1040 procedure [24] at a final concentration of 5 ppm with the adjusted cell suspension. Survivors were counted after incubation by comparing the number of colonies with those obtained when biocide was replaced by distilled water. Each experiment was done in triplicate.

In parallel, the susceptibility to peracetic acid at 5 ppm of planktonic suspensions of *B. subtilis* 168, *B. subtilis* ND_medical_ and *S. aureus* AH478 was evaluated. The resistance of *S. aureus* AH478 suspension in absence or presence of extracellular matrix extracted from *B. subtilis* 168 or ND_medical_ biofilm was also determined. In this aim, biofilm matrix extracts were directly added to *S. aureus* AH478 suspension at a 1/10 (vol/vol) concentration before disinfection step according to the NF EN 1040 standard procedure. Finally, we determined the susceptibility of mixed suspensions of *S. aureus* with *B. subtilis* 168 or ND_medical_ strain to evaluate to potential role of a soluble effector in the resistance of the pathogen in mixed communities. In this end, *S. aureus* AH478 and *B. subtilis* 168 or ND_medical_ were grown together in TSB overnight before peracetic acid treatment at 5 ppm using NF EN 1040 standard procedure.

#### Real-time visualization of PAA activity in biofilms

Time lapse CLSM analysis of PAA action was performed in *B. subtilis* biofilms as previously described [25]. This technique permits the direct visualization of cell inactivation patterns in biofilm structures during biocide action. The method is based on the monitoring of fluorescence loss caused by the leak of an unbound fluorophore outside the cells after disruption of the bacterial membrane by antimicrobial agents. Experimentally, 24 h biofilms in a microtiter plate were rinsed with TSB in order to eliminate planktonic cells. The wells were then refilled with 100 µl of TSB containing Chemchrome V6 (1∶100 of commercial solution (AES Chemunex, France)). Chemchrome V6 is an esterasic marker which enter cells passively and is then cleaved by cytoplasmic esterases, leading to the intracellular release of fluorescent residues (green fluorescence). Microtiter plates were incubated in the dark at 20°C for 1 h in order to reach fluorescence equilibrium. Biofilms were then rinsed to eliminate excess Chemchrome V6 and refilled with 100 µl of TSB. The microtiter plate was then mounted on the motorized stage of a Leica TCS SP2 AOBS Confocal laser scanning microscope (LEICA Microsystems, France) at the MIMA2 microscopy platform (http://voxel.jouy.inra.fr/mima2). The CLSM control software was set to take a series of time-lapse xyzt scans (256×256 pixels) at intervals of 15 s at five different depths in the biofilm. Biofilms were scanned at 400 Hz using a 63× oil objective with a 1.4 numerical aperture with a 488 nm argon laser set at 10% of its maximal intensity. These settings, which were shown to avoid photobleaching of the sample by performing preliminary scans using distilled water instead of biocides, were used in all time-lapse experiments. Emitted V6 Chemchrome green fluorescence was recorded within the range 500–600 nm. After the starting of the xyzt scan series, 100 µl of PAA at 7000 ppm were gently added to the wells just after the completion of the first scan. Biofilms were then scanned every 15 s at five different depths over 10 min, and fluorescence loss within the structure was recorded. Three independent experiments were performed for each strain.

Quantification of green fluorescence intensity from the time-lapse (xyzt) CLSM image series was performed using confocal software LCS Lite (Leica Microsystems, France). Intensity values were then normalized by dividing the fluorescence intensity recorded by the initial fluorescence intensity value at the same location. Fluorescence intensity curves shown are the mean of three experiments, each experiment constituting the average of five horizontal sections along the depth of the biofilm.

#### Application of bacterial destruction models on fluorescence intensity curves

GinaFiT, a freeware Add-inn for Microsoft® Excel developed by Geeraerd *et al.* [26] was used to modelize inactivation kinetics. This tool enables testing of nine different types of microbial survival models, and the choice of the best fit depends on five statistical measures (i.e., sum of squared errors, mean sum of squared errors and its root, R^2^, and adjusted R^2^). During this study, the “log-linear + tail” inactivation model was fitted on fluorescence intensity curves obtained from CLSM image series during biocide treatment. The inactivation rate k_max_ (min^−1^) was extracted from the fitting.

#### Detection of spores

In order to check the presence of spores in the biofilm after 24 h of development, cells were recovered from the microtiter plates as described above and resuspended in 150 mM NaCl and boiled at 100°C for 10 min. Bacterial numbers (cfu/ml) were determined before and after the heat treatment.

### Statistical Analysis

One-way ANOVA was performed using Statgraphics v6.0 software (Manugistics, Rockville, USA). Significance was defined as a *P* value associated with a Fisher test value lower than 0.05.

## Results

### Phenotypic Characterization of Biofilms Formed by ND_medical_ and 168 Strains

**Figure 1:**
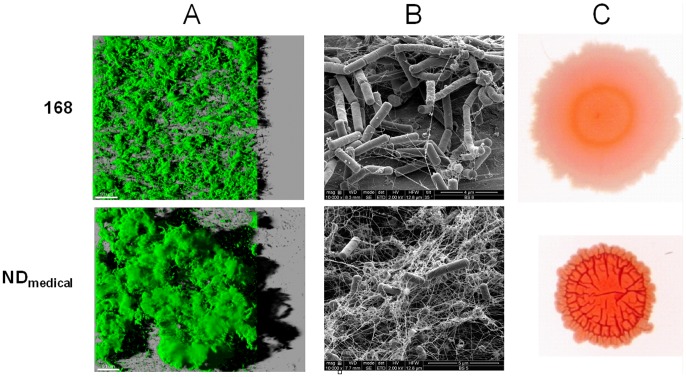
Architecture of *B. subtilis* communities. (A) Three-dimensional projection of *B. subtilis* 168 GFP and ND_medical_ GFP biofilms obtained from xyz confocal images series using IMARIS software. Images present an aerial view of biofilm structure with the virtual shadow projection on the right. Scale bar correspond to 50 µm. (B) SEM images of 24 h-biofilms. (C) Dye binding properties of *B. subtilis* macrocolonies grown for 72 h on Congo red indicator plates.

Biofilms formed by a non-domesticated *B. subtilis* strain (ND_medical_) recently isolated from an endoscope washer-disinfector and by the laboratory *B. subtilis* 168 strain were characterized. First, the architecture of biofilms formed by both *B. subtilis* GFP strains was observed after 24 h of development using CLSM as presented in Fig. 1A. The images correspond to three-dimensional reconstructions obtained from confocal stack images by the IMARIS software, including virtual shadow projections on the right. In accordance with previously published observations [17], we found that the non-domesticated *B. subtilis* ND_medical_ strain clearly developed a thicker biofilm with protruding structures in comparison to strain 168 in these experimental conditions.

In order to visualize the extracellular material produced in biofilms of both *B. subtilis* 168 and the ND_medical_ strains, SEM observations were also performed. In both cases, the extracellular material detected appeared to be composed of tangled thread-like strands forming a complex network. However, this was clearly much denser in the biofilm of the ND_medical_ strain as shown in Fig. 1B. It should be noted here that SEM observations required several dehydration steps during sample preparation and therefore the electron micrographs can not be considered as faithful representations of fully hydrated biofilms. However, these results clearly indicated the presence of larger amount of extracellular substances in ND_medical_ strain biofilm in comparison to strain 168.

Since Congo Red is known to bind extracellular substances, it has been widely used in previous works to compare the ability of different strains to produce extracellular components in numerous bacterial species including *B. subtilis* [27], [28], [29]. Macrocolonies of both *B. subtilis* strains grown for 72 h on Congo Red indicator plates are presented in Fig. 1C. We observed that ND_medical_ produced highly wrinkled macrocolonies as compared to strain 168. In addition, the ND_medical_ strain stained red more intensively on Congo Red indicator plates, suggesting a higher amount of exopolymeric substances.

In line with this, biochemical quantification of the extracellular matrix confirmed that ND_medical_ produced more abundantly extracellular components than strain 168. Indeed, sugar and protein contents of biofilm matrix reached respectively 33.5±1.4 and 26.7±1.0 mg/l for ND_medical_ strain whereas these values attained only 13.1±0.8 and 8.7±0.3 mg/l for the strain 168.

### *B. subtilis* ND_medical_ Biofilms Exhibit a Marked Resistance to PAA

The resistance of biofilms formed by both *B. subtilis* strains exposed to 3500 ppm of PAA for 5 min was then investigated. These conditions represent the *in-use* PAA concentration and time of exposure commonly used in hospitals for the disinfection treatment of endoscopes. The results showed substantial resistance of the *B. subtilis* ND_medical_ strain which exhibited only 3.8 log of reduction while no survivor were detected on plates for strain 168, corresponding to a reduction greater than 5.6 log (Table 2). Using thermic treatment, we confirmed that ND_medical_ biofilm did not contain any spores at 24 h of development showing that the resistance observed was displayed by vegetative cells.

**Table 2:** Bactericidal activity of water and 0.35% PAA on single and mixed species biofilms after 5 min of treatment.

		log (CFU/well)	
	Strain	Water	PAA (0.35%)
**Single species biofilm**	*B. subtilis* 168	7.6±0.2	–
	*B. subtilis* NDmedical	7.7±0.1	3.9±0.6
	*S.aureus* AH478	9.3±0.1	–
**Mixed species biofilm**	*B. subtilis* 168	7.5±0.5	–
	*S.aureus* RN4220	8.2±0.4	–
	*B. subtilis* NDmedical	7.3±0.3	3.9±0.3
	*S.aureus* RN4220	8.4±0.1	2.6±0.5

Data shown are mean of three experiments ± standard deviation. Samples from which no survivor were recovered are represented by (−). Minimum detection of 2 logs CFU/well.

**Figure 2:**
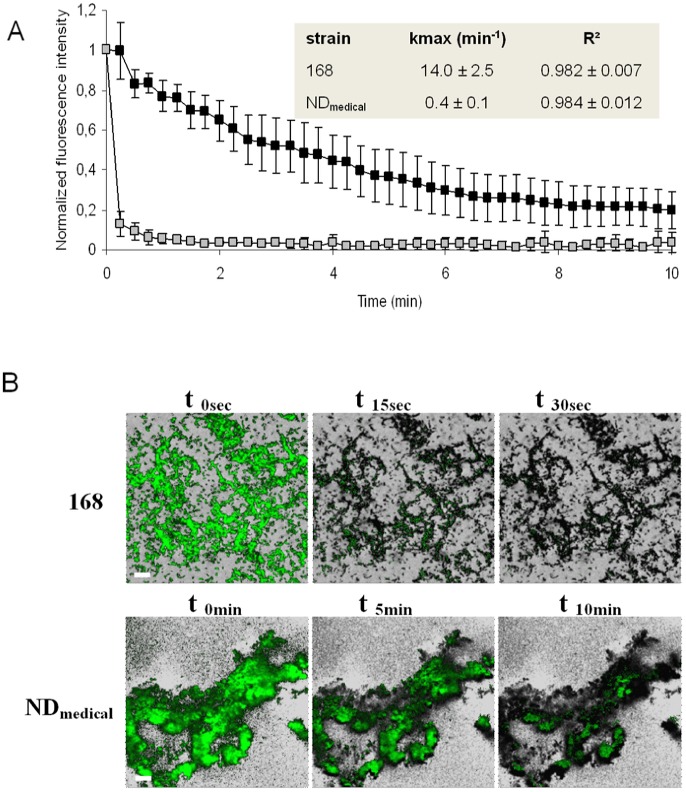
Real-time visualization of PAA (0.05%) activity in B. subtilis biofilms. (A) Quantification of Chemchrome V6 fluorescence intensity in B. subtilis 168 (white squares) and ND_medical_ (black squares) biofilms during PAA treatment at 500 ppm. Values shown corresponds to the average of the fluorescence at five depths with standard deviation in the biofilm for a representative experiment. Inactivation rate k_max_ was obtained after fitting GinaFIT “log linear + tail” inactivation model on experimental data. The means of three experiments ± standard deviation are presented in the inset. (B) Visualization of Chemchrome V6 fluorescence loss (membrane permeabilisation) in B. subtilis 168 and ND_medical_ biofilms at 0. 15 and 30 secondes and 0. 5 and 10 minutes respectively during PAA treatment (0.05%). Each image corresponds to the superimposition of a green fluorescence image of a representative experiment on a greyscale image of initial fluorescence at the same location. Scale bars correspond to 20 µm.

The resistance of the ND_medical_ biofilm was investigated further by time-lapse CLSM microscopy allowing to visualize biocide action in real-time within the biofilm structures of both *B. subtilis* strains. Figure 2A presents the dynamics of fluorescence loss (membrane permeabilisation) within the biofilms during treatment with 500 ppm of PAA. This concentration was chosen in order to enable the visualization of biofilm damage in approximately 10 min. When biofilms were treated with distilled water as a control, the loss of fluorescence observed was less than 1% (n = 4) of the initial fluorescence after 10 min of treatment. The GinaFIT “log linear + tail” inactivation model [26] was applied to these experimental data to extract the inactivation rate k_max_ and this revealed satisfactory adjustment (R^2^>0.982). The results confirmed the greater resistance of the biofilm for strain ND_medical_ as compared to the laboratory strain 168. While PAA treatment resulted in an immediate and uniform loss of fluorescence of cells in the biofilm for strain 168, the loss of fluorescence in the ND_medical_ biofilm was slow and heterogeneous during disinfection treatment. The extracted k_max_ inactivation rates showed a large and significant difference between strain 168 and ND_medical_ (inset in Fig. 2, *P*<0.05) with values of 14.0 and 0.4 respectively. Spatial patterns of fluorescence loss in biofilms treated with 500 ppm PAA are presented in Figure 2B. A homogeneous and sharp loss of fluorescence was seen in the whole biofilm structure of strain 168 following the application of biocide. Conversely, PAA application led to a non-homogenous loss of fluorescence within the biofilm structure of the ND_medical_ strain. Indeed, with this strain, we observed that spreading of PAA was difficult in some areas and these remained fluorescent even after 10 min of treatment, illustrating the high resistance and associated low inactivation rate k_max_ displayed by this strain.

The resistance to PAA (5 ppm) of cells following removal and washing from the biofilm of strain ND_medical_ was investigated and compared to the resistance of the corresponding planktonic cells. Reductions of 2.9±0.3 and 2.8±0.1 log (cfu/ml) were obtained for planktonic cells and detached cells respectively *i.e.* no significant difference (*P*>0.05). The disruption of the biofilm and at least partial elimination of extracellular substances thus led to the disappearance of the PAA resistance.

### The ND_medical_ Strain Provides Protection to *S. aureus* Against PAA in a Mixed Biofilm

The previous results showed that the ND_medical_ biofilm exhibited marked resistance to PAA treatment and suggested that the extracellular matrix likely plays an important role in this resistance. We next investigated whether the ND_medical_ biofilm can provide protection in a mixed biofilm to *S. aureus*, a pathogenic strain present in medical environments and often incriminated in nosocomial infections.

**Figure 3:**
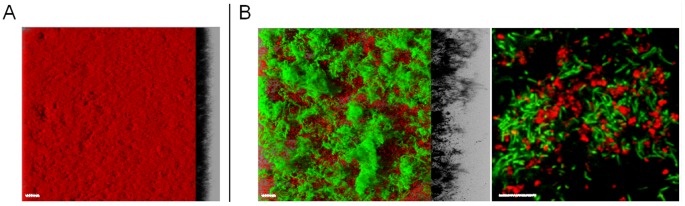
Architecture of *S. aureus* AH478 mCherry biofilm and *B. subtilis* ND_medical_ GFP/*S.aureus* AH478 mCherry mixed biofilm. (A) 3D reconstruction of *S.aureus* AH478 mCherry biofilms.(B) 3D reconstruction and section at higher magnification of mixed species biofilm of *B. subtilis* ND_medical_ GFP (green)/*S.aureus* AH478 mCherry (red). Scale bars correspond to 20 µm.

As shown on Fig. 3A, *S*. *aureus* alone produced flat and regular biofilm with a thickness of 20–30 µm. When this single species biofilm was exposed to 3500 ppm of PAA for 5 min, the small numbers of survivors on plates corresponded to a more than 7.3 log of reduction compared to distilled water control (Table 2). However, when growing in a mixed biofilm with *B. subtilis* ND_medical_, the same PAA treatment produced a 5.9 log reduction of *S. aureus* suggesting that pathogenic strain acquired benefit from strain association. Note that *B. subtilis* ND_medical_ resistance to PAA was similar in both single and mixed biofilms with 3.8 and 3.4 log reduction respectively. In contrast, mixed biofilm composed of the lab strain *B. subtilis* 168 and *S. aureus* AH478 did not gain a higher resistance to PAA compared to the single species biofilms.

Confocal observations showed that, in the presence of *S. aureus* AH478, *B. subtilis* ND_medical_ was able to develop three-dimensional structures similar to those observed in the single species biofilm of this strain (Fig. 3 B). The *S. aureus* cells largely covered the surface of the bottom of the wells not occupied by the *B. subtilis* strain although some cells were trapped in the protruding structures formed by the ND_medical_ strain.

Interestingly, when *B. subtilis* ND_medical_ biofilm matrix extract was added to a *S. aureus* AH478 suspension before peracetic treatment at 5 ppm, we enumerated survivors on Petri dishes with a reduction of 3.6±0.3 log whereas no survivor (log reduction >4.1 log) was observed when matrix extract from *B. subtilis* 168 biofilm was added to *S. aureus* AH478 suspension in the same conditions. When examining the resistance of mixed overnight culture of *S. aureus* AH478 with each *B. subtilis* strains to peracetic acid, we did not observed, in both cases, a significant better resistance of the pathogen compared to single *S. aureus* culture (*P*>0.05).

Together, these results underline that a direct interaction of ND_medical_ biofilm matrix components with peracetic acid likely plays a key role in the better resistance of *S. aureus* AH478 observed in mixed biofilm.

## Discussion

In a previous study, Martin *et al*. [16] isolated a strain of *B. subtilis* (called ND_medical_ in this study) from an endoscope washer-disinfector and showed that this strain developed a high resistance against various oxidising agents using a standard suspension test. On the basis of SEM observations, they suggested that this resistance could be due to a higher production of matrix in comparison with sensitive strains. Recently, we showed that this stable isolate produces a very thick biofilm with specific protruding and elevated structures in comparison to other *B. subtilis* strains [17], and this was confirmed in our present observations. As biofilm formation is frequently associated with increased resistance to sanitizing treatments and antibiotic therapies [30], [31], [32], the enhanced biofilm-forming ability of strain ND_medical_ can thus represent a strategy to survive despite the regular exposure to biocide treatments. In line with this, Shemesh *et al.* [4] showed that sublethal doses of chlorine actually stimulated biofilm formation by *B. subtilis*, reflecting an apparent defensive response against biocide action. In addition, it has been shown in *P. aeruginosa* that hyper-biofilm forming variants appear due to self-generated diversity occuring in the biofilm, and this was correlated with higher resistance of the whole community to hydrogen peroxide [33]. Our present results showed a very high resistance of the ND_medical_ biofilm to PAA, an oxidizing agent commonly used in formulations used in endoscope disinfection; highlighting that biofilm formation could indeed enable this strain to persist despite drastic disinfection treatments applied during endoscope decontamination. Some evidence appears to suggest that this high resistance could be mainly related to an increased production of extracellular matrix in agreement with the previous observations made by Martin *et al.* [16] with planktonic cells. First, the disruption of the three-dimensional biofilm structure and washing of cells led to the restoration of a PAA sensitivity similar to that observed for planktonic cells. In addition, SEM observations and Congo red assays showed that the ND_medical_ strain produced a denser and more abundant extracellular substances when compared with strain 168, which was much more sensitive to PAA. Finally, biochemical assay showed that sugars and proteins contents in ND_medical_ biofilm were respectively 2.6 and 3.1 times higher than in the biofilm formed by the strain 168. These results highlight the primordial role of the three-dimensional structure of the biofilm of ND_medical_ strain and the abundant matrix that could play a protective role through the interaction with and the quenching of PAA reducing thus its penetration into the biofilm. Previous studies reported that penetration limitations encountered by chlorine, another oxidising agent, in biofilms of different species can explain at least partly the reduced activity of this molecule probably due to interactions with biofilm constituents [34], [35], [36]. Kinetic profiles and spatial patterns of fluorescence loss (cell membrane permeabilization) obtained using CLSM time lapse visualization of PAA action in the ND_medical_ biofilm (Fig. 2) were consistent with these observations. Indeed, the progressive action of PAA in the heterogeneous structure of biofilm, with cells in some internal areas remaining fluorescent even after 10 min of treatment illustrate the limited action of the biocide in these areas. In line with these observations, we can hypothesize that the presence of dense matrix domains in the ND_medical_ biofilm may constitute the principal mechanism involved in resistance to PAA. Whether this phenotype is related to one or several matrix components needs to be addressed. In particular, the involvement of molecular constituents already known to participate to the matrix composition of *B. subtilis* such as *epsA-O* operon products [37], [38] or TasA amyloid fibers [29], [39] for the most clearly identified, should be investigated. The nucleotide sequences of the *epsA-O* and *yqxM/sipW/tasA* operons, including their promoter regions, were identical in both the ND_medical_ and 168 strains (data not shown). In addition, construction of transcriptional fusion genes with a fluorescent reporter associated with flow-cytometry showed no significant difference between expression of the *epsA-O* operon in NDmedical and 168 strains, whereas expression of the *yqxM/sipW/tasA* operon was significantly higher in biofilms of the reference strain as compared to the NDmedical strain (*P*<0.05, data not shown). Taken together, these data indicated that the resistance observed of the strain ND_medical_ was likely not due to modification of any of these major elements involved in the *B. subtilis* matrix composition, but rather to different genes that remain to be identified.

We also investigated whether *S. aureus* can be protected from disinfection in a mixed biofilm with *B. subtilis* ND_medical_ strain. *S. aureus* is a bacterial pathogen commonly found in hospitals and responsible for a large number of infections [18], [40], and numerous studies have reported the presence of *S. aureus* on endoscopes even after disinfection procedures [15], [41]. Our results demonstrated that more than 7.3 log of reduction (no survivor enumerated) were obtained for *S. aureus* single species biofilm after PAA treatment at 3500 ppm whereas we found only 5.9 log of reduction when growing in mixed biofilm with ND_medical_ strain (Table 2). These results showed a protective effect of the presence of *B. subtilis* ND_medical_ strain to *S. aureus* against PAA activity. Confocal images revealed that some *S. aureus* cells were entrapped in the protruding structures formed by the ND_medical_ strain and thus probably benefited from the protective matrix of the *B. subtilis* strain. Supporting this hypothesis,addition of *B. subtilis* ND_medical_ biofilm matrix extract to a *S. aureus* AH478 planktonic suspension led to a better resistance of the pathogen to peracetic acid. Moreover, since the concomitant growth of *S. aureus* with the ND_medical_ strain in suspension had no effect on the resistance of *S. aureus*, it is likely that the higher survival of *S. aureus* in mixed biofilm was mainly due to the quenching of the oxidizing agent by the abundant extracellular matrix produced by the *B. subtilis* ND_medical_ strain, rather than a soluble effector which could trigger the pathogen resistance. Different works already reported that species associations in mixed biofilms can result in a higher resistance to disinfection compared to single species biofilms [42], [43], [44], [45], [46] and that some species can protect another throughout interspecies spatial associations. Leriche *et al.* [47] reported for instance that *Staphylococcus sciuri* was protected in a mixed biofilm against a chlorinated solution by microcolonies formed by *Kocuria* sp., a more resistant strain. As it is clear that microbial communities present in medical or industrial areas are complex multispecies associations rather than controlled single species model, these findings highlight the importance to challenge biocides on representative multispecies biofilm models of the environment considered. In this study, peracetic acid alone was tested but it should be highlighted that further studies are required to understand the impact on PAA-containing formulations. Product formulations can vary considerably on their activity against biofilms due to presence of other chemicals, such as chelating agents and detergents. These can help detach and disaggregate the biofilms, thus facilitating biocidal activity.

In conclusion, this work points out that the selection pressure maintained in healthcare environments by regular disinfection treatments can lead to the emergence of strains with hyper-resistance phenotypes. This is related to the great ability of bacteria to adapt to their environments by developing efficient survival strategies such as biofilm formation. Particular attention should thus be paid to the choice of strains which are representative of the reality of medical areas in disinfectant testing. In addition, the necessity to consider the resident microbial ecosystem (including non-pathogenic but resistant strains as *B. subtilis* ND_medical_) instead of pathogen alone is emphasized since the persistence of a strain can be related not directly to its intrinsic resistance but also to the interactions with other species present in the environment. Future works will focus on the understanding of the adaptation mechanisms leading to an increased resistance of bacteria to biocides in these environments.
